# Antimicrobial Peptides: Bringing Solution to the Rising Threats of Antimicrobial Resistance in Livestock

**DOI:** 10.3389/fvets.2022.851052

**Published:** 2022-04-08

**Authors:** Shamsaldeen Ibrahim Saeed, AhmedElmontaser Mergani, Erkihun Aklilu, Nor Fadhilah Kamaruzzaman

**Affiliations:** ^1^Faculty Veterinary Medicine, University Malaysia Kelantan, Pengkalan Chepa, Malaysia; ^2^Faculty of Veterinary Science, University of Nyala, Nyala, Sudan; ^3^Department of Biochemistry, University of Veterinary Medicine Hannover, Hanover, Germany; ^4^Research Center for Emerging Infections and Zoonoses (RIZ), University of Veterinary Medicine Hannover, Hanover, Germany; ^5^Department of Microbiology, Faculty of Veterinary Medicine, University of Khartoum, Khartoum North, Sudan

**Keywords:** antimicrobial peptides (AMPs), livestock, antimicrobial resistance (AMR), bacterial infection, alternative antimicrobial

## Abstract

Antimicrobial therapy is the most applied method for treating and preventing bacterial infection in livestock. However, it becomes less effective due to the development of antimicrobial resistance (AMR). Therefore, there is an urgent need to find new antimicrobials to reduce the rising rate of AMR. Recently, antimicrobial peptides (AMPs) have been receiving increasing attention due to their broad-spectrum antimicrobial activity, rapid killing activities, less toxicity, and cell selectivity. These features make them potent and potential alternative antimicrobials to be used in animals. Here, we discuss and summarize the AMPs in animals, classification, structures, mechanisms of action, and their potential use as novel therapeutic alternative antimicrobials to tackle the growing AMR threat.

## Introduction

Livestock infectious disease represents a big threat to animal health, welfare, public health, environment, and food security (1). It can cause huge economic impacts due to the increased mortality, cost of treatment and control, reduced productivity, loss in trade, and decreased gross domestic product (GDP) (2). The treatment and control of livestock diseases is crucial for livestock industries, safeguarding public health, and securing global food supplies. Antimicrobials are commonly used for the treatment and prevention of bacterial infection in livestock. These practices, in part, are associated with increased rates of antimicrobial resistance (AMR) among pathogenic bacteria isolated from animals (3). There is rising concern that overuse of antimicrobials has led to the emergence of resistant organisms to most or all antimicrobials and thus can lead to therapeutic failure (4). Resistant bacteria can spread from food animals to humans through different routes including direct contact with livestock, indirect contact through food consumption, and animal waste used as a fertilizer of crops and can contaminate water supplies (5, 6). Therefore, there is an increasing need to evaluate and develop alternative methods for antimicrobial treatment (7). A wide range of alternative antimicrobial approaches have been developed by researchers worldwide to find effective methods to tackle the infection caused by AMR. These methods include the application of antimicrobial peptides (AMPs) (8–12).

AMPs are gene-encoded polypeptide sequences that are considered essential elements of immune defenses in all organisms (13). The antibacterial properties of AMPs observed in *in vitro* conditions have long attracted the attention of scientists looking to address AMR's problem. Both natural and synthetic AMPs have proved strong and broad-spectrum antibacterial activity *in vitro* and efficacy in various animal infection models. At the same time, their actions are considered unaffected by canonical bacterial resistance mechanisms that reduce the effectiveness of conventional antibiotics (14). Besides the direct antimicrobial activity, AMPs has an anti-biofilm effect by suppressing biofilm formation and destabilizing the biofilm structure (13). In addition, AMPs have immunomodulatory effects by stimulating the immune response. The versatile role of AMPs is highlighted not only in eliminating pathogens but also in boosting immunity to induce better protection against infection (15). Therefore, the AMPs have potential for the development of novel alternative antimicrobials to replace the existing antibiotics. This review will summarize and discuss the recent update with AMPs in animals and their mechanisms of antimicrobial activity. Also highlighted is the therapeutic application of novel alternative antimicrobials for the treatment of bacterial infection in livestock.

## Antimicrobial Peptides (AMPs)

AMPs are small-sized proteins that are crucial elements in host immune defense in most living organisms, including animals, humans, insects, fish, and plants (16). AMPs are short-chain amino acids (composed of 10–50 amino acids), amphiphilic, and positively charged (17). This feature allows them to bind and penetrate the bacterial membrane bilayer to induce pores by “toroidal-pore”, “barrel-stave,” and “carpet”, thus causing intracellular leakage (16). AMPs have received a great amount of attention due to their broad spectrum of antimicrobial activity against various microorganisms such as fungi, viruses, and bacteria in both veterinary and human pathogens (18), rapid killing activities, less toxicity, and cell selectivity (18, 19). In addition, a new study found that the combination of AMPs with conventional antibiotic increased the efficacy in killing bacteria and preventing the development of AMR. These findings highlight the potential adjunctive administration of AMPs in clinical applications (20).

## Classification of AMPs

**Figure 1:**
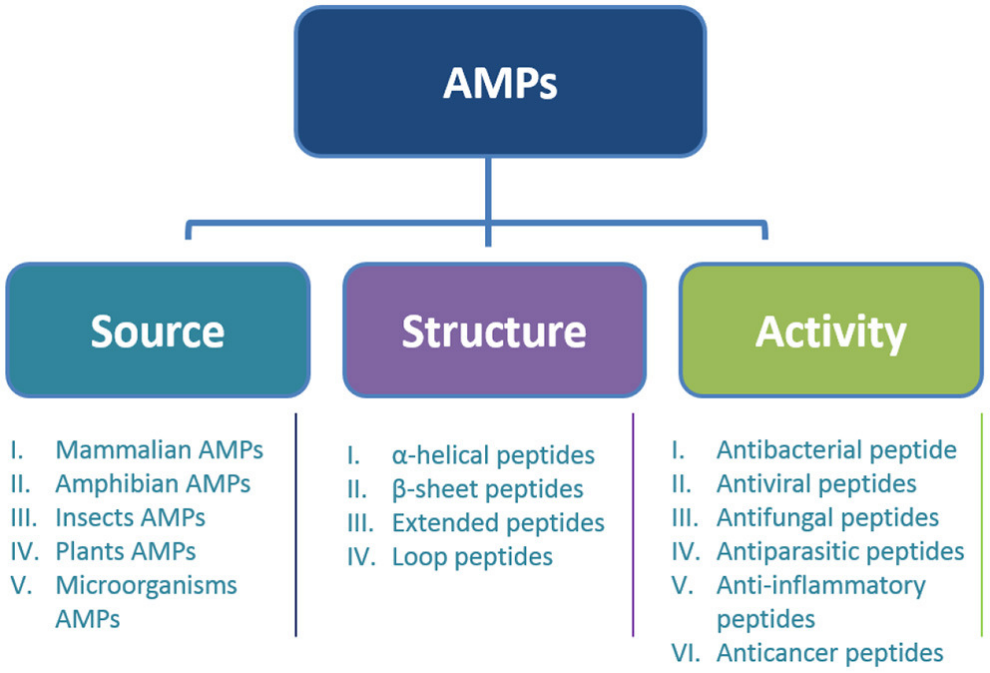
Classification of AMPs.

AMPs are large and diverse and can basically be divided into major groups based on structure, source, and biological activity (20) (Figure 1). Based on the sources, the peptide can be found in animals, humans, insects, microorganisms, and plants, and based on structure, the AMPs can be divided into four categories: α-helix, β-sheet, extended, and loop (9). On the other hand, based on biological activity, peptides can be divided into antibacterial peptides, antifungal peptides, antiviral peptides, antiparasitic peptides, and anticancer peptides (21).

### Animal AMPs

Animals possess a wide range of AMPs that protect them against infection. Some of these peptides have been fully studied and characterized, and most of them have activity against diverse pathogens. The mammalian AMPs can be divided into two major classes: cathelicidins and defensins (21). AMPs from animals can be found in many parts of their bodies such as skin, intestine, blood, saliva, and milk. The most AMPs from animals include indolicidin, Bovine Psoriasin, Bovine Defensin 1, Protegrin, Cecropin P1, Ovine Defensins, and lactoferrecin. Table 1 summarizes the major AMPs. These peptides play an essential role in host immunity and protect them from infection (22). Dairy is a rich source of AMPs; many peptides have been identified from casein, lactoglobulin, and lactoferrin, among them is lactoferrecin, considered the most common peptide found in milk (23). Some of these AMPs such as indolicidin have been approved to be used in the treatment of bovine mastitis (24), while the majority of AMPs are still in preclinical studies.

**Table 1:** Summary of some selected AMPs and their mechanisms of action.

**Antimicrobial peptides**	**Source**	**Activities**	**Mechanism of action**	**References**
Plectasin	Fungi	Gram-positive bacteria	Interrupting cell wall biosynthesis	(11)
Nisin	Bacteria	Gram-positive bacteria	Pore formation in the bacterial cell membrane and interrupting cell wall biosynthesis	(25)
Peptaibols	Fungi	Fungi and bacteria	Permeabilize bacterial membrane	(26)
Protegrin	Porcine lung and intestine	Gram-negative, gram-positive bacteria, and yeast	Pore formation in the bacterial cell membrane and immunomodulation	(27)
PR-39	Porcine intestine, upper and lower respiratory tract	Gram-negative bacteria and *Mycobacterium tuberculosis*	It inhibits protein and DNA and synthesis by exerting proteolytic activity and acts as a calcium-dependent chemoattractant for neutrophil	(28, 29)
SMAP29	Ovine myeloid cells	Gram-negative, gram-positive bacteria, and yeast	Permeabilize bacterial membrane	(30)
Bovine lactoferrcin (lfcin)	Bovine	Bacteria, fungi, virus, and parasite	Bind and realized LPS from bacteria and disruption the cell membrane	(31)
Indolicidin	Mammalian	Gram-positive and Gram-negative bacteria, yeast, and fungi	Membrane thinning, disruption of the membrane by channel formation, inhibition of DNA synthesis, and topoisomerase 1	(30–32)
Bovine Psoriasin	Bovine	Gram-negative bacteria	Reduces bacterial survival by zinc sequestration	(33)
Buforin 2	Amphibia	Fungi, Gram-positive, and Gram-negative bacteria	Targeting the biosynthesis of RNA	(34)

### Amphibian AMPs

AMPs from amphibians have a vital role in the protection of amphibians from the invasion by pathogenic microorganisms (22). Certain amphibians can produce AMPs, and frogs are considered a major source of amphibian peptides (35). Magainins and dermaseptins are two classes of cationic, amphipathic alpha-helical peptides that have been identified in the skin extracts of frogs *Phyllomedusa sauvagei* and *Xenopus laevis* (36). Magainins are 23-amino-acid-long peptides that belong to a large family of amphibian amphipathic α-helical AMPs. These peptides have been reported to have a wide spectrum of antimicrobial activities against Gram-negative and Gram-positive bacteria and fungi, and mechanisms of action due to the disruption of the bacterial membrane (20). On the other hand, these peptides are also reported to have a cytotoxic effect toward mammalian cells at 31.25 μg/ml (20), but they did not cause red blood cell (RBC) hemolysis up to 300 μg/ml (37). Dermaseptins are linear polycationic peptides, composed of 28–34 amino acids, which are structured in amphipathic-helices in apolar solvents (38). These peptides demonstrated broad-spectrum antimicrobial activity, including multidrug-resistant strains. In addition, dermaseptins have been found to be less cytotoxic compared to magainins (20).

### Insect AMPs

Insects are one of the biggest sources of AMPs (39). Several AMPs are produced by insects, and these AMPs are mainly found in blood cells or in fat tissues of insects and help in survival mechanisms to protect the insect from diseases (22). The most common insect AMPs are defensins, cecropins, ponericins, drosomycin, drosocin, attacins, diptericins, and metchnikowin (37). Cecropins are effective against both Gram-negative and Gram-positive bacteria, whereas defensins can selectively kill Gram-positive bacteria only (38). Most insect AMPs have strong antimicrobial activities and low toxicity, making them excellent candidates to be developed as alternative antimicrobials.

### Plant AMPs

Plants are known to have several AMPs, and it is reported that they have potent antimicrobial activity against pathogenic bacteria, viruses, and parasites besides having anticancer and anti-inflammatory activity (21). Most AMPs from plants share the same feature as those from animals, insects, and microorganisms. For example, defensins, thionins, and cyclotides are the common plant AMPs, and they look similar to AMPs from animals in terms of amphipathic nature, molecular form, positive charge, and Cys-rich peptides (39, 40). On the other hand, some plant peptides are different from those from animals such as hevein-like peptides that bind chitins (40). Currently, thousands of plant AMPs have been identified and none of them has been approved yet for clinical use.

### Microorganism AMPs

AMPs can be obtained from microorganisms such as fungi and bacteria. Some common peptides are bacteriocins derived from lactic acid bacteria such as *Lactococcus lactis, Bacillus brevis*, and *Bacillus subtilis* (21). The most common bacteriocin includes nisin, mersacidin, lacticin 481, and lacticin 3147. Among them, nisin has been approved for commercial use for the treatment of bovine mastitis (25), while mersacidin and lacticin have promising results for the treatment of infection caused by bacteria, particularly antibiotic-resistant bacterial strains such as methicillin-resistant *Staphylococcus aureus* (MRSA) and vancomycin-resistant enterococci (VRE) (41). Plectasin is another APM that belongs to defensin from microorganisms isolated particularly from the fungus saprophytic ascomycete *Pseudoplectania nigrella*. Plectasin demonstrated strong bactericidal activity against Gram-positive bacteria such as *S. aureus* and particularly multidrug-resistant strains with extremely low toxicity (42).

## Structural Classes of AMPs

**Figure 2:**
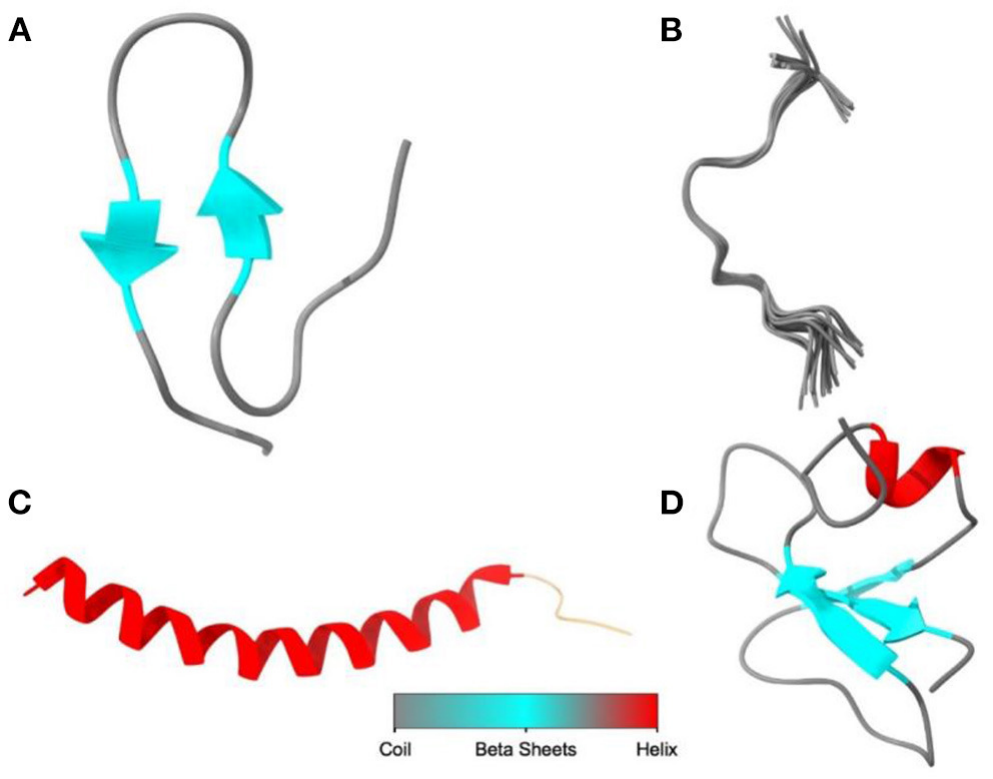
The diagram illustrated the main structural classes of AMPs: **(A)** β-sheet, defensins, and protegrins; **(B)** extended, indolicidin; **(C)** α-helical, nisin, and lactoferricin; **(D)** loop or combined structure, plectasin. The image was created using UCSF Chimera (http://www.cgl.ucsf.edu/chimera).

AMPs are generally divided into four major classes based on their structure: α-helical, β-sheet, extended, and loop peptides (17) (Figure 2). Both natural and synthesis peptides shared the same structure, and the most common feature for all peptide groups is the ability to fold into amphiphilic or amphipathic conformation due to their interaction with membrane (17). The α-helical peptides are the most abundant class of natural peptides in nature. These peptides will, upon interaction with target membranes fold into an amphipathic α-helix. “Amphipathicity” is the property of having hydrophobic and hydrophilic regions separated in space” with one face of the helix predominantly containing the hydrophobic amino acids and the opposite face containing the charged amino acids (43). β-sheet peptides are indicated by the presence of an antiparallel β-sheet stabilized by two or more disulfide bonds (17) and include, among others, the defensins of vertebrates, insects, and plants (44). Extended peptides such as indolicidin contain high proportions of certain amino acids such as tryptophan, histidine, and proline (45). Most of these peptides adopt extended structures upon interaction with the membrane, and this is stabilized by hydrogen bonds and van der Waals forces with lipids rather than interresidue hydrogen bonds (46). Loop peptides are characterized by their loop structure imparted by the presence of a single bond, i.e., disulfide and amide (17).

## Antimicrobial Mechanisms of AMPs

The antimicrobial mode of action of AMPs is complicated, and one single peptide can target different sites in the microbe (47). The antimicrobial activity of AMPs is believed to be due to inhibition of cell wall, nucleic acid and protein synthesis, and inhibition of enzymatic activity (21, 47). The antimicrobial activity of AMPs is particularly linked to its corresponding amino acid composition and physicochemical characteristics (45). Several studies have identified that the antimicrobial mechanism of AMPs kills bacteria due to increased membrane permeability, induction of lipid asymmetry, and loss of cellular components and essential metabolites, which ultimately leads to cell death (47–49). In addition to membrane permeabilization, AMPs can kill bacterial cells by targeting not only DNA but also the biosynthesis of cell wall, LPS, and other biological pathways (50).

### Membrane Interaction Mechanisms

**Figure 3:**
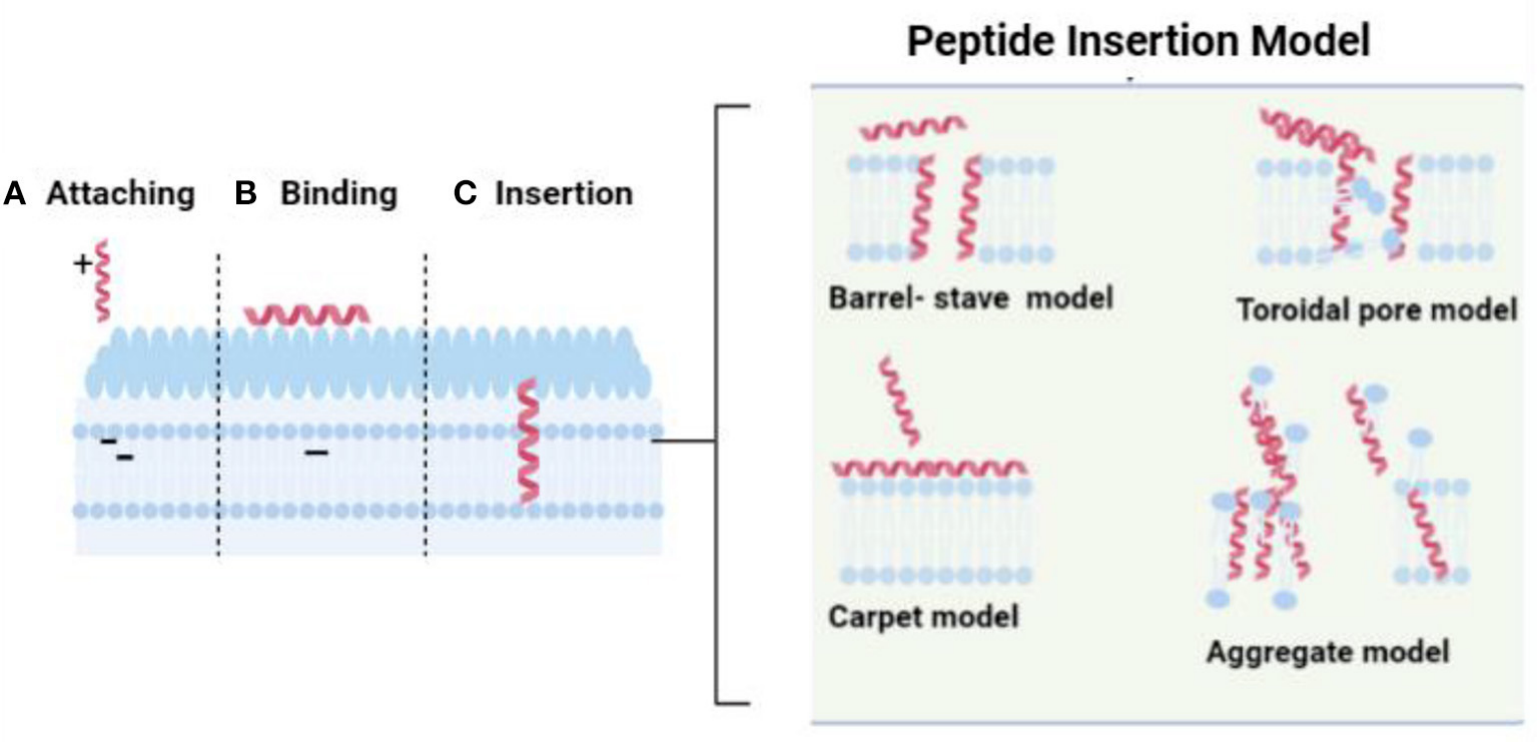
The interaction between peptide and bacterial cellular membrane. The image was created using BioRender illustrator (https://Biorender.com/).

AMPs attach to the bacteria cell wall by electrostatic interactions between the anionic component of a membrane and the positive charge of a peptide (21, 51, 52). After binding, the peptides cross the cell wall and cell membrane to contact the cellular membrane in Gram-positive bacteria. For Gram-negative bacteria, the first action of AMPs involves the competitive displacement of Mg2+ and Ca2+. In this way, peptides destabilize this supramolecular assembly and gain access to both inner and outer membranes. Following the attachment, AMPs are inserted into the membrane to form transmembrane pores and are divided into four models: (1) the barrel-stave model in which the peptides penetrate the membrane and form pores in the hydrophilic portion, (2) the carpet model in which the peptides disrupt the membrane structure by a detergent-like action, (3) the toroidal model in which the hydrophilic portion of the amphipathic conformation of peptides is associated with the lipid headgroup, and (4) the aggregate model in which the peptide penetrates the membrane and damaging it (21) (Figure 3).

### Targeting Intracellular Components

**Figure 4:**
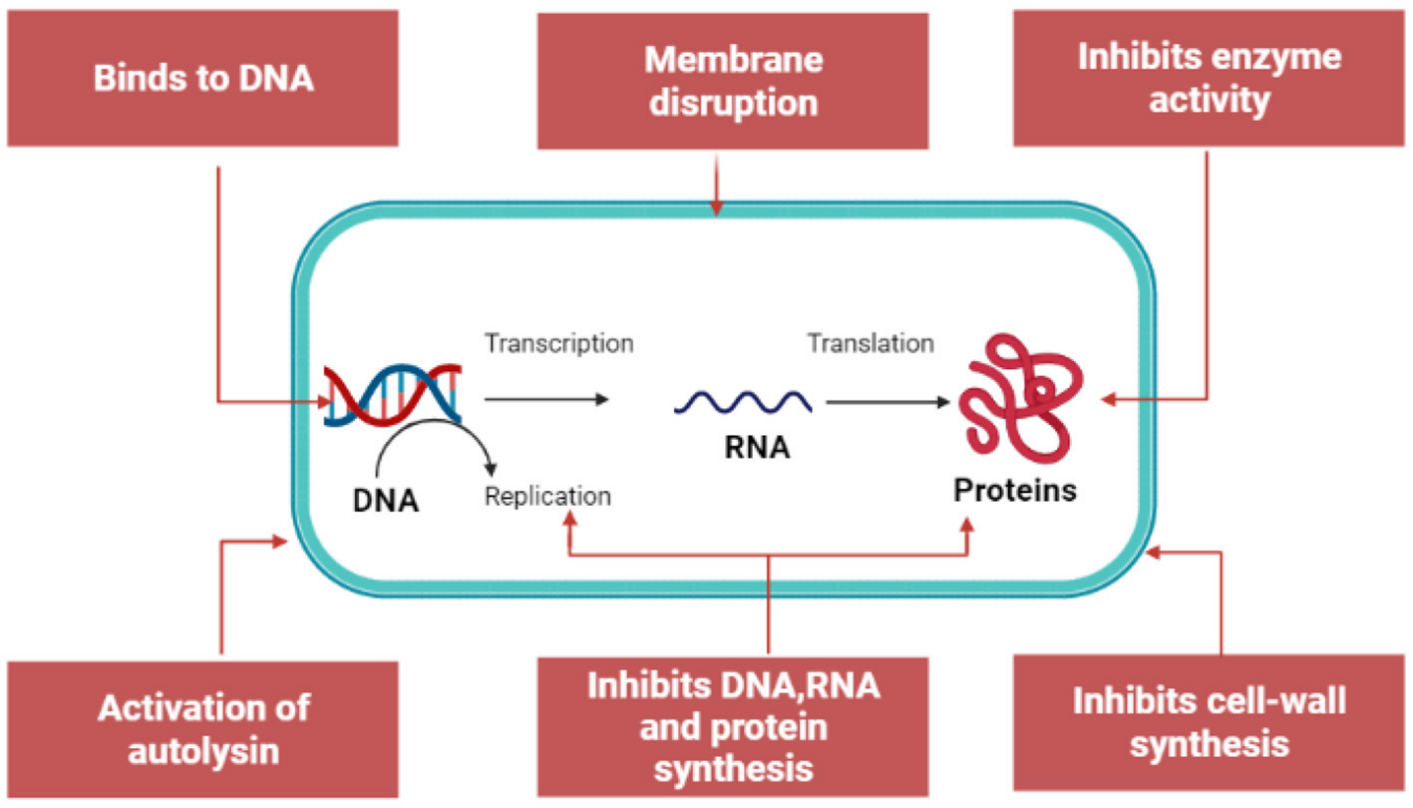
Mechanism for intracellular antimicrobial peptide activity. The image was created using BioRender illustrator (https://Biorender.com/).

Besides the membrane damage, the peptide can kill bacteria by inhibiting the biosynthesis of nucleic acid, proteins, and some essential enzymes from synthesizing cell walls and bacteria growth (21). Figure 4 summarizes the mechanisms for the intracellular AMPs. AMPs can interfere with key enzymes involved in transcription, translation, and assembly, such as chaperones, leading to inhibition of proteins. For example, pyrrhocoricin targets ribosomes to inhibit protein translation, whereas PR-39 inhibits protein synthesis in *E. coli* by inhibiting the transition from the initial to the extension phase (34, 53).

Furthermore, AMPs can induce degradation of DNA and RNA or affect key enzymes involved in DNA synthesis. For example, indolicidin can target a basic site of DNA to crosslink single- or double-stranded DNA, and it can also inhibit DNA topoisomerase I (54, 55). Besides inhibiting nucleic acids and proteins, AMPs can inhibit the metabolic activity of cells due to the effect on protease activity (21).

### Recent Advances in the Use of AMPs for the Treatment of Bacterial Infection in Animals

Peptide-based antimicrobials have shown efficacy towards pathogenic bacteria in animals. Some of them such as nisin were approved for clinical use while others are at the advanced stages of clinical trials (Table 2). Several studies have reported that AMPs have strong antibacterial activity against a wide range of pathogens. Tomasinsig et al. (24) found that the cathelicidin family of peptide from bovine such as BMAP-27, BMAP-28, Bac5, and indolicidin has a broad spectrum of activity against most bacterial isolates from bovine mastitis with MIC in the range of 0.5–32 μM. Besides the mastitis in cattle, AMPs particularly lactoferricin (lfcin) and nisin have demonstrated strong antibacterial, anti-fungal, and antiparasitic activity with potential use in both animals and humans for the treatment of infection (31, 56).

**Table 2:** Antimicrobial activity of the peptide-based antimicrobial compound against pathogenic bacteria isolated from livestock.

**Peptide**	**Bacteria spp**.	**MIC range (μM)**	**References**
Plectasin	*S. aureus*	3–6	(11)
Nisin	*S. epidermidis* *S. aureus*	30 >32	(56)
Lactoferrcin (lfcin)	*S. aureus* *S. epidermidis* *E. coli* *Listeria monocytogenes*	20-100 10–20 13–167 2–20	(31)
Indolicidin	*E. coli* *K. pneumoniae* *S. aureus* *S. epidermidis* *S. uberis* *S. agalactiae*	4 4–8 2–8 1–2 1–2 1–2	(24)
Cathelicidins Bac5	*E. coli* *K. pneumoniae* *S. aureus* *S. epidermidis* *S. uberis* *S. agalactiae*	0.5–1 1–4 >32 1–2 16–32 4–6	(24)
Cathelicidins BMAP-28	*E. coli* *K. pneumoniae* *S. aureus* *S. epidermidis* *S. uberis* *S. agalactiae*	2–8 1–2 2–4 1–2 2–32 2	(24)
Cathelicidins BMAP-27	*E. coli* *K. pneumoniae* *S. aureus* *S. epidermidis* *S. uberis* *S. agalactiae*	0.5–4 1 4–8 0.5–1 4 4	(24)

### Plectasin

**Figure 5:**
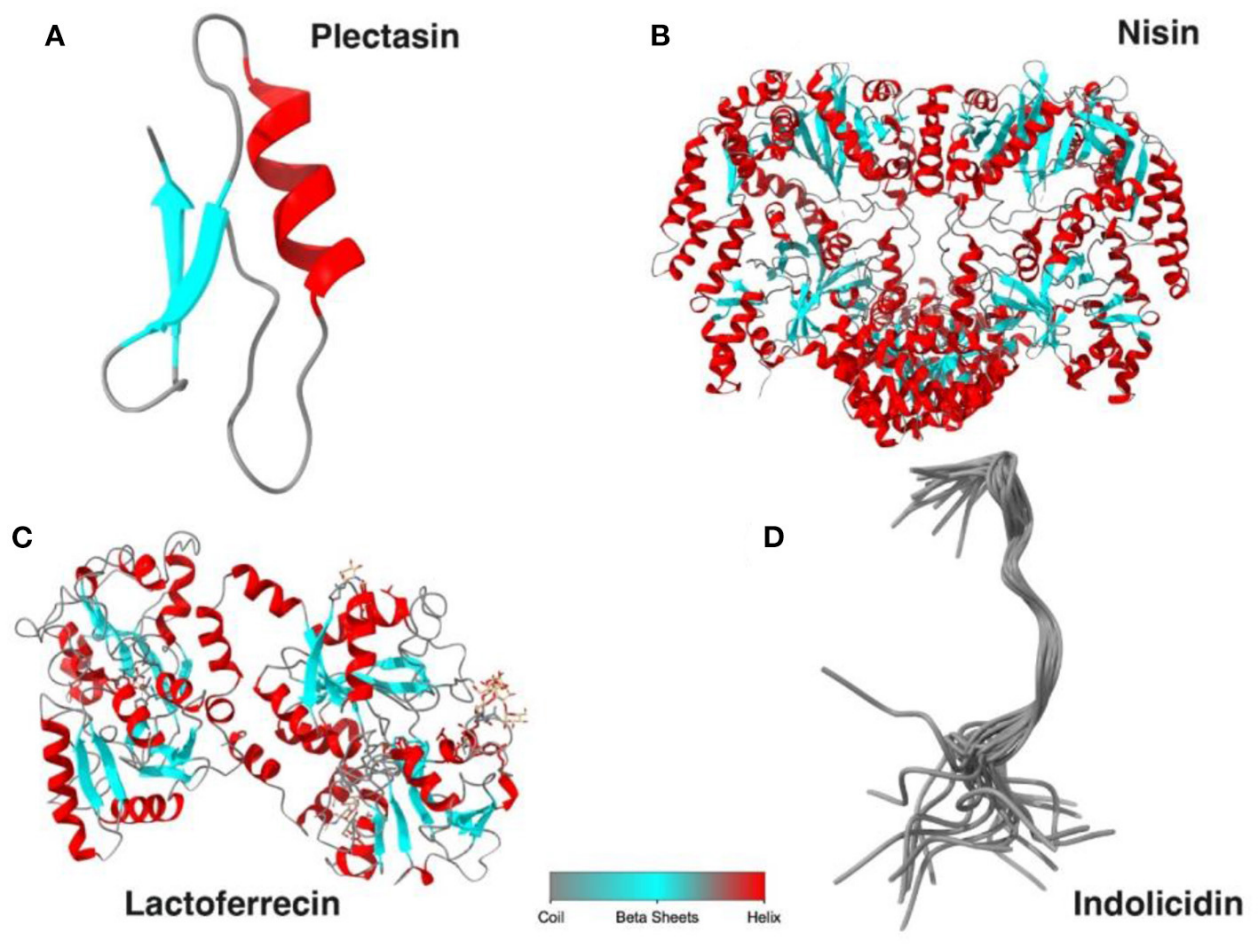
The diagram illustrates the peptide structure for plectasin **(A)**, nisin **(B)**, lactoferricin **(C)**, and indolicin **(D)**. The image was created using UCSF Chimera (http://www.cgl.ucsf.edu/chimera).

Plectasin is a peptide-based antimicrobial (Figure 5A) first isolated from the saprophytic ascomycete *Pseudoplectania nigrella*, and it was reported to have strong antimicrobial activity (11, 42). Plectasin peptide can kill bacteria through interference with cell wall biosynthesis by specifically binding to Lipid II, which is the vital element of bacterial cell wall precursor (57). Despite the strong antimicrobial activity, plectasin was reported to have low toxicity toward mammalian cells, making them a strong candidate as an alternative antimicrobial (12). In a recent study conducted by Li et al. (11), they found that plectasin-derived AMPs such as NZ2114 and MP1102 have strong bactericidal activity toward *S. aureus* mastitis with MIC of 1–2 μg/ml in media. In addition, the plectasin AMPs demonstrated potency against intracellular *S. aureus* at 100 μg/ml, reducing 100% of *S. aureus* inside the mammary epithelial cells.

### Nisin

Nisin is an AMP belonging to the lantibiotic family (Figure 5B) that has strong antimicrobial activities against Gram-positive bacteria (56). This peptide is commonly used as a food preservative, and it is approved by FDA and WHO. Nisin is produced by the bacteria *Lactococcus lactis* with 34 amino acids. It is considered as a promising alternative antimicrobial to replace existing antibiotics, particularly for the treatment of infections caused by multidrug-resistant bacteria. Nisin displays bactericidal properties due to it binding to lipid II in bacteria membrane, leading to inhibition of cell wall biosynthesis and induced form in bacterial cell membrane (56, 58). Recently, nisin is developed and formulated as the product to be used clinically to treat mastitis, and it shows promising results. For example, the study conducted by Cao et al. reported that treatment of bovine mastitis with intramammary administration of 2,500,000 IU resulted in a 90.2% clinical cure rate. In addition, all *S. aureus* isolated from mastitis were sensitive toward nisin, while 82.5% of them were resistant to penicillin and 35.3% were resistant to gentamicin (42).

### Lactoferrecin

Lactoferrecin is an iron-binding glycoprotein peptide derived from lactoferrin F (Figure 5C). Besides its host immune defense, this molecule displays strong antimicrobial activity against bacteria, virus, fungi, and parasite and can be an excellent alternative antimicrobial to combat many diseases such as mastitis (31). The bactericidal effect of lactoferricin is believed to be due to its ability to disrupt microbe cellular permeability and inhibition of cell wall due to electrostatic interaction between the positive charge in amino acids such as arginine in peptide and the negative charge in bacteria membrane such as LPS in Gram-negative and lipoteichoic and teichoic acids in Gram-positive bacteria. On the other hand, lactoferricin also displays anticancer activity and low toxicity toward mammalian cells (59). In addition, lactoferricin is considered an excellent alternative drug for mastitis treatment due to its activity against different mastitis pathogens. In the study, Kawai et al. examined the efficacy of these compounds toward subclinical mastitis caused by *Staphylococci* and *E. coli*. The study recorded the decrease in bacteria load on the first day of treatment and a 100% cure rate from mastitis after 14 days (60).

### Indolicidin

Indolicidin is an extended cationic antimicrobial peptide and is a member of the cathelicidin group with 13 amino acids, rich in tryptophan and proline, and is amidated at the carboxyl terminus in nature (32) (Figure 5D). It was initially purified from the cytoplasmic granules of neutrophils from bovine (61). It can also be synthesized in bone marrow cells as a 144-aa-long precursor (62). Indolicidin demonstrated antimicrobial activity against several microorganisms including *Candida albicans, Cryptococcus neoformans, S. aureus*, and *E. coli*, and its antimicrobial action includes disruption of the bacterial membrane by channel formation and inhibition of DNA replication (8, 63). The membrane damage exhibited by indolicidin could be due to its optimum hydrophobicity along with the pore formation in bacterial membrane resulting in membrane lipid–bilayer partition (64, 65). In the recent study conducted by Vergis (66), they found that indolicidin was effective against multidrug-resistant enteroaggregative *E. coli* (MDR-EAEC) with a MIC of 32 μM, and at 2 × MIC and 4 × MIC, clearance of MDR-EAEC was completed after 120 min of coincubation. This bacterial elimination by AMPs highlighted the potency of the peptide compared to conventional antibiotics.

### Cathelicidins

Cathelicidins are short cationic peptides that are part of the innate immune system, and they are found in mammals, including humans (24). These peptides demonstrated broad-spectrum activity against microorganisms. In addition, they can protect against infection by modulating other components of the innate or adaptive immune response (24, 67, 68). Cathelicidins belong to the family of host defense peptides (HDPs), with each cathelicidin encoded by a single gene, consisting of four exons (69). Bovine cathelicidins BMAP-27, BMAP-28, and Bac5 produced by myeloid-derived cells are well studied for their antimicrobial activity (24). For example, a study conducted by Tomasinsig et al. (24) found that the AMPs from family cathelicidins showed a potent antibacterial activity toward bacteria isolated from bovine mastitis. The tested peptides include BMAP-27, BMAP-28, and Bac5 with a MIC value of 0.5–32 μM, and the mechanisms of antimicrobials are due to disruption of bacterial membrane integrity. In addition, the study also found that these peptides were also able to effectively trigger expression of the proinflammatory mediator TNF in bovine mammary epithelial cells and stimulate the immune response to act against invading bacteria (24).

## Conclusions

Antimicrobial agents serve as an important component in the treatment and control of bacterial infection in livestock. However, the treatment becomes ineffective due to the development of AMR, leading to therapeutic failure. Therefore, there is an urgent need to find alternatives to existing antimicrobials. Recently, AMPs have been explored and introduced as a novel alternative antimicrobial due to their unique antimicrobial properties in target bacteria, fungi, viruses, and parasites; AMPs also have anticancer activity and an immunomodulatory effect. In addition, AMPs are considered less toxic to mammalian cells. However, there is limited information regarding the antimicrobial application of AMPs in animals. In this review, we have summarized the importance of antimicrobials, classification, and source and mechanism of peptides' antimicrobial activity. In addition, recent advances in AMP use for the treatment of bacterial infection in livestock are highlighted. AMPs demonstrated promising results in the treatment of bacterial infection, particularly the AMR strain. Therefore, AMPs could be an excellent alternative antimicrobial to be used as replacement for existing antibiotics.

## Author Contributions

SS designed the framework of the review, provided the illustration, and wrote the first draft. AM contributed to manuscript writing. EA and NK supervised the work and editing and revised the final version of the manuscript for publication. All authors read and approved the final manuscript for publication.

## Funding

This work was supported by the Fundamental Research Grant Scheme by Malaysia Ministry of Higher Education grant code FRGS/1/2017/SKK11/UMK/03/1.

## Conflict of Interest

The authors declare that the research was conducted in the absence of any commercial or financial relationships that could be construed as a potential conflict of interest.

## Publisher's Note

All claims expressed in this article are solely those of the authors and do not necessarily represent those of their affiliated organizations, or those of the publisher, the editors and the reviewers. Any product that may be evaluated in this article, or claim that may be made by its manufacturer, is not guaranteed or endorsed by the publisher.
